# A multicenter descriptive analysis of anemia management in hemodialysis patients and its association with quality of life

**DOI:** 10.1186/s12882-023-03254-7

**Published:** 2023-06-30

**Authors:** Samah W. Al-Jabi, Nada S. Rajabi, Amer A. Koni, Sa’ed H. Zyoud

**Affiliations:** 1 Department of Clinical and Community Pharmacy, College of Medicine and Health Sciences, An-Najah National University, Nablus, 44839 Palestine; 2 Division of Clinical Pharmacy, Department of Hematology and Oncology, An-Najah National University Hospital, Nablus, 44839 Palestine; 3 Poison Control and Drug Information Center (PCDIC), Faculty of Medicine and Health Sciences, An-Najah National University, Nablus, 44839 Palestine; 4 Clinical Research Center, An-Najah National University Hospital, Nablus, 44839 Palestine

**Keywords:** Anemia, Management, Hemodialysis, Hemoglobin, Quality of life, Erythropoietin-stimulating agent

## Abstract

**Background:**

Appropriate management of anemia in patients with hemodialysis (HD) involves the administration of iron supplementation and erythropoietin-stimulating agents (ESAs), in addition to monitoring the response. This study aimed to evaluate the treatment of anemia in patients with HD and describe the factors associated with it and its effect on health-related quality of life (HRQOL).

**Methods:**

The study was cross-sectional in design. The patients were included from three dialysis centers in Palestine from June to September 2018. The data collection instrument consisted of two portions; the initial portion contained demographic and clinical information on the patients, while the second consisted of the European Quality of Life 5-Dimension Scale (EQ-5D-5 L) and the visual analog scale EQ (EQ-VAS).

**Results:**

The study included 226 patients. Their mean age (± SD) was 57 ± 13.9 years. The mean level of hemoglobin (Hb) (± SD) was 10.63 ± 1.71 g/dl, and 34.1% of the patients had a Hb level of 10-11.5 g/dl. All patients who required iron supplementation received it intravenously with a dose of 100 mg of iron sucrose. Almost 86.7% of the patients received darbepoetin alfa intravenously at 0.45 mcg/kg a week, and 24% had a Hb level > 11.5 g/dl. There were significant associations between the level of Hb and the number of comorbid diseases and the ESA that was received. However, other demographics and clinical factors did not significantly affect Hb levels. Certain variables, such as exercise, were a predictor of a higher quality of life. It should be noted that there is a significant impact of a low Hb value on the EQ-VAS scale.

**Conclusions:**

Our study found that more than half of the patients had a Hb level below the recommended goal of Kidney Disease Improving Global Outcomes (KDIGO). Furthermore, a significant association was found between patients’ Hb level and HRQOL. Therefore, the appropriate treatment of anemia in patients with HD should be followed by adherence to the guideline recommendations, which consequently improves the HRQOL of HD patients, in addition to obtaining optimal therapy.

**Supplementary Information:**

The online version contains supplementary material available at 10.1186/s12882-023-03254-7.

## Background

Hemodialysis (HD) is the backbone and life-sustaining therapy for patients with end-stage renal disease (ESRD) who cannot undergo renal transplantation [1–3]. The reported number of patients with ESRD who underwent HD in the West Bank increased from 1014 to 2015 to 1119 patients in 2016, divided into 12 units [4, 5]. The hemodialysis procedure can cause frequent complications related to ESRD [6]. Anemia is a frequent complication among ESRD patients, affecting almost all HD patients [7, 8].

Anemia contributes to mortality and morbidity [9], adverse cardiovascular outcomes, and a lower quality of life (HRQOL) [7]. Although erythropoietin-stimulating agents (ESAs) are available, the management of anemia in patients who receive renal replacement therapy is controversial [9, 10].

The most inclusive objective for managing patients with ESRD is maintaining a long survival rate and preserving the patient’s life at a satisfactory level [11]. Good anemia management positively affected HD patients, including lowering mortality and morbidity rates with a substantial improvement in HRQOL [12–14]. In particular, HRQOL deterioration has increased over time, and one of its anticipated factors was changes in hemoglobin (Hb) [15]. Furthermore, previous studies have suggested a correlation between HD, anemia, and reduced HRQOL [15, 16].

A previous study found that 45% of patients with HD had a mean Hb level between 11.0 and 12.0 g/dl, which was according to the KDOQI recommendation, 30% were between 10.0 and 11.0 g/dl, 10% had a level lower than 10.0 g/dl, and 15% were above 12.0 g/dl. The study also showed that women and patients with a low albumin level had a statistically significant low Hb level [17]. The European Survey on Anemia Management 2003 (ESAM 2003) [18] has conducted a study in 11 European countries. The results revealed that diabetic nephropathy is the most verified reason for renal failure, hypertension is the communal comorbidity, and the majority of precipitants had received HD. Furthermore, the proportion of patients who reached a target Hb level of ≥ 11 g//dl varied between countries [18]. Data from a prospective and multicenter observational study demonstrated that the mean Hb level was 10.29 ± 1.44 g/dl, and 26% of the patients had Hb levels between 11 and 11.9 g/dl [14]. A cross-sectional study exhibited a significant positive correlation between Hb and hematocrit levels and HRQOL [16].

In Palestine, an observational retrospective study assessed compliance with the management guidelines among HD patients. As a result, 8.9% and 43% of the patients achieved the Hb goals according to the National Kidney Foundation Kidney Disease Outcomes Quality Initiative (NKF-KDOQI) and KDIGO, respectively. Additionally, the study found an insignificant association between anemia control and sociodemographic and clinical factors of patients [19].

Treatment of anemia in ESRD is challenging for physicians and patients [14], as inappropriate treatment is associated with poor results and reduced HRQOL. Few articles have been published to evaluate anemia management among HD patients and their correlation with HRQOL [16, 20–22], and none of these studies were conducted in Palestine. The main goal of the current study was to evaluate the treatment of anemia among patients with maintenance HD. Furthermore, we aimed to describe the characteristics of the study patients associated with anemia and to assess the association between anemia and HRQOL.

Our study is the first in Palestine to focus on this issue. Therefore, this research provides a framework and information that can be a reference to improve anemia management. Additionally, determining factors associated with a low Hb level among HD and evaluating the treatment strategy can help improve the treatment approach of these patients.

## Methods

### Study design

This study was cross-sectional in design, achieved using a convenient and clustered sampling technique. Participants were recruited from three dialysis centers in the north, center and south West Bank, Palestine: An-Najah National University Hospital in Nablus, Ramallah’s Sons Ward of Palestine Medical Complex in Ramallah, and Al Hussein Government Hospital in Beit Jala.

### Sample size

In 2016, the West Bank, under the Ministry of Health, operated a total of 11 hemodialysis units, which were equipped with 148 hemodialysis machines. Furthermore, An-Najah National University Hospital in Nablus had one unit with 35 hemodialysis machines. As a result, the West Bank was able to offer consistent dialysis services to 1,119 patients [5]. During that year, the estimated population of the region was approximately 3 million, and the prevalence of hemodialysis cases was 373 per million population.

According to the annual health report for Palestine 2016, the number of patients who underwent dialysis in the three centers was 484 [5]. This number was used to calculate the required sample size using the Roasoft tool (http://www.raosoft.com/samplesize.html), with a response distribution of 50%, a 95% confidence interval, and a margin of error of 5%. The calculated minimum effective sample size was 215. Furthermore, the estimated sample size was increased by 5-10% to improve the reliability of the present study and decrease false results.

An-Najah National University Hospital received 47.8% (108 patients) of the sample, Ramallah’s Sons Ward received 32.7% (74 patients) and Al Hussein Government Hospital received 19.5% (44 patients). Furthermore, a pilot study of 10 patients was conducted before starting the actual study, which was not included in the analysis.

### Data collection

Data were collected from the three dialyzes from June to September 2018. The questionnaire was completed throughout the working time of each center by interviewing the enrolled patients for almost 10 min and accessing their profiles.

The questionnaire consisted of two parts. The first part contained the patient’s sociodemographic and clinical information divided into the following: [1] Sociodemographic characteristics, including age, gender, level of education (illiterate, primary school, secondary school, or university), marital status (married or single), location (urban, rural, or camp), employment status (unemployed, employed, or previously employed before the onset of renal failure), smoking (current smoker, previous smoker, or nonsmoking), exercise, herbal use, and family history [2]. Clinical information on the history of the disease and other chronic diseases included the number of HDs per week, the duration of renal failure, the duration of HD and the presence of comorbidities [3]. Current medications, herbal products used, and last available laboratory values.

The second part consisted of the European Quality of Life 5-Dimensions scale (EQ-5D). The Euro Quality of Life (QOL) Group developed the EQ-5D instrument, consisting of the descriptive system and the visual analog scale (EQ-VAS). The descriptive system contains five components (mobility, usual activities, self-care, anxiety/depression, and pain/discomfort). Furthermore, EQ-VAS records the respondent’s self-rated health on a 20 cm vertical visual analog scale with endpoints labeled ‘the best health you can imagine’ and ‘the worst health you can imagine’. EQ-VAS records how good or bad a patient’s health status is today by marking ‘X’ on the scale and then writing the number in the box below. The Arabic form of the EQ-5D has been used in several populations [23–27] and, more specifically, in Palestine HD patients [28–32]. In accordance with the recommendations of the tool’s developers [33], the Arabic version of the EQ-5D was used with permission. The EQ-5D scale had good and acceptable internal consistency, with a Cronbach alpha score of 0.820.

### Inclusion and exclusion criteria

ESRD patients who were on maintenance HD, 18 years and older, and accepted participation were included. However, we excluded patients who were unable to understand the question, such as psychiatric patients, cancer patients, or actively receiving chemotherapy.

### Ethical considerations

Approvals from the Institutional Review Board (IRB), the Palestinian Ministry of Health (PMOH), and An-Najah National University Hospital were obtained before the initiation of the current investigation. Furthermore, verbal consent was obtained from patients individually, and only patients who agreed to participate were included.

### Statistical analysis

The data were analyzed using version 21 of the Statistical Package for Social Sciences (IBM-SPSS) program. The analysis involved presenting the data using the mean (± standard deviation), median (lower-upper quartile), frequencies, and percentages. To assess normality, the Kolmogorov‒Smirnov test was employed. Correlations between the categories of Hg level and other factors with the quality of life scales were examined using the Kruskal‒Wallis and Mann‒Whitney U tests. The EQ-5D-5 L crosswalk index value calculator, based on values from the general population of the United Kingdom, was used to determine the EQ-5D scores. Differences in categorical variables were evaluated using the chi-square or Fisher exact test. Multiple linear regression analysis was conducted to identify the determinants of poor HRQOL. The significance level was set at a p-value of less than 0.05.

## Results

### Characteristics of the sample

A total of 226 HD patients were included in the current analysis. The mean age (± SD) was 57 ± 13.9 years, ranging from 21 to 87 years. More than half of the patients (*n* = 121, 53.5%) were male. The highest proportion (*n* = 117, 51.8%) of patients lived in rural areas, followed by urban areas (*n* = 86, 38.1%). The marital status reported by the patients was as follows: 78.8% were married, and 21.2% were single/divorced/widowed. Regarding the level of education, the majority (*n* = 86, 38.1%) of the patients had secondary education, followed by (*n* = 80, 35.4%) a primary education. A small proportion (*n* = 13, 5.8%) of the patients reported having a current job, while 124 (54.9%) were previously employed. The majority (*n* = 136, 60.2%) of the patients were nonsmokers, and only 7.5% exercised (Table 1).

**Table 1:** Characteristics of the study sample according to hemoglobin level

**Characteristic**	Total (N = 226)	Hb ˂ 11 g/dl	Hb ≥ 11 g/dl	P value
	**n (%)**			
**Gender**				0.119
Male	121 (53.5)	66 (49.3)	55 (59.8)	
Female	105 (46.5)	68 (50.7)	37 (40.2)	
**Age category (years)**				0.279
< 60	113 (50.0)	71 (53.0)	42 (45.7)	
≥ 60	113 (50.0)	63 (47.0)	50 (54.3)	
**Locality**				0.391
Rural	117 (51.8)	53 (39.6)	33 (53.9)	
Urban	86 (38.1)	65 (48.5)	52 (56.5)	
Camp	23 (10.2)	16 (11.9)	7 (7.6)	
**Marital status**				0.61
Married	178 (78.8)	104 (77.6)	74 (80.4)	
Single	48 (21.2)	30 (22.4)	18 (19.6)	
**Level of education**				0.95
Illiterate	19 (8.4)	12 (9.0)	7 (7.6)	
Primary	80 (35.4)	47 (35.1)	33 (35.9)	
Secondary	86 (38.1)	52 (38.8)	34 (37.0)	
University	41 (18.1)	23 (17.2)	18 (19.2)	
**Employment status**				0.555
Unemployed	89 (39.4)	55 (41.0)	34 (37.0)	
Employed	13 (5.8)	9 (6.7)	4 (4.3)	
Previously employed before renal failure	124 (54.9)	70 (52.2)	54 (58.7)	
**Smoking**				0.249
Current smoker	52 (23)	33 (24.6)	19 (20.7)	
Previous smoker	38 (16.8)	18 (13.4)	20 (21.7)	
Nonsmoker	136 (60.2)	83 (61.9)	52 (56.5)	
**Exercise**				0.324
No	209 (92.5)	122 (91)	87 (94.6)	
yes	17 (7.5)	11 (8.2)	5 (5.4)	
**Family history**				0.501
No	187 (82.7)	109 (81.3)	78 (84.8)	
Yes	39 (17.3)	25 (18.7)	14 (15.2)	
**Herbal use**				0.285
No	103 (45.6)	65 (48.5)	38 (41.3)	
Yes	123 (54.4)	69 (51.5)	54 (58.7)	
**Total number of medications**				0.16
< 7	115 (50.9)	63 (47.0)	52 (56.5)	
≥ 7	111 (49.1)	71 (53.0)	40 (43.5)	
**Number of dialysis per week**				0.939
< 3	25 (11.1)	15 (11.2)	10 (10.9)	
≥ 3	201 (88.9)	119 (88.8)	82 (89.1)	
**Duration of renal failure**				0.425
< 5	124 (54.9)	73 (54.5)	51 (55.4)	
5-<10	72 (31.9)	46 (34.3)	26 (28.3)	
≥ 10	30 (13.3)	15 (11.2)	15 (16.3)	
**Years of hemodialysis**				0.209
< 4	146 (64.6)	91 (67.9)	55 (59.8)	
≥ 4	80 (35.4)	43 (32.1)	37 (40.2)	
**Hours of hemodialysis**				0.429
< 4	158 (69.9)	91 (67.9)	67 (72.8)	
≥ 4	68 (30.1)	43 (32.1)	25 (27.2)	
**Number of comorbid diseases**				**0.045**
≤ 2	32 (14.2)	14 (10.4)	18 (19.6)	
3–4	97 (42.9)	56 (41.8)	41 (44.6)	
5–6	75 (33.2)	46 (34.3)	29 (31.5)	
> 6	22 (9.7)	18 (13.4)	4 (4.3)	
**Received darbepoetin alfa**				**< 0.001**
No	30 (13.3)	6 (4.5)	24 (26.1)	
Yes	196 (86.7)	128 (95.5)	68 (73.9)	

**Abbreviation**: IQR: Interquartile range, p value: probability value, Hb: hemoglobin

Regarding comorbid diseases, the median number (interquartile range) was 4.0 (3.0-5.1). Most patients suffered from hypertension (87.6%), followed by diabetes mellitus (DM) (56.2%) and ischemic heart disease (42.5%) (Additional file 1: Table S1).

### History of renal disease of the study sample

The median duration (interquartile ranges) (years) of renal failure and HD of the study participants was 4.0 (2.0–6.0) and 2.5 (1.0–5.0), respectively. Furthermore, the median number (interquartile range) of HD sessions per week was 3.0 (3.0–3.0), and the median number of hours (interquartile range) of HD sessions was 3.5 (3.5-4.0).

### Chronic medications and herbal remedies used by the study sample

The results showed that 123 patients (54.4%) used herbal remedies. Arabic gum (*n* = 71, 31.4%) was the most popular herb used, followed by mixed herbs (*n* = 42, 18.6%) (Additional file 1: Table S2).

The number of medications used was between 0 and 14, with a median (interquartile range) of 6.0 (5.0–8.0). Darbepoetin alfa (*n* = 196, 86.7%), calcium carbonate (*n* = 193, 85.4%), alfacalcidol (*n* = 190, 84.1%) and iron sucrose (*n* = 128, 56.6%) were the most commonly used medications (Additional file 1: Table S3).

### Laboratory values for the study sample

The mean Hb level (± SD) was 10.63 ± 1.71 g/dl, ranging from 6.10 to 17.31 g/dl. According to the definition of anemia by KDIGO, 109 (90.1%) male patients were anemic with Hb ˂ 13 g/dl, and 83 (79%) female patients were anemic with Hb ˂ 12 g/dl. Indeed, 84 (37.16%) patients had Hb values < 10 g/dl, 134 (59.3%) patients had Hb values ˂ 11 g/dl, 92 (40.7%) had Hb values > 11 g/dl, and 77 (34.1%) had Hb values 10-11.5 g/dl.

Table 2 shows a variety of laboratory results obtained from patient medical records. Of 226 patients, 128 (56.6%) received intravenous iron therapy. The 128 patients received iron sucrose at a dose of 100 mg. Information on iron status was available for 163 (72.1%) patients with recorded serum ferritin and 185 (81.90%) with documented TSAT. The mean TSAT (± SD) of the patients was 32.33 ± 17.16%, ranging from 5.41 to 130, and the mean (± SD) serum ferritin level was 447.49 ± 464.45 ng/ml, ranging from 4.63 to 2016. Regarding the KDIGO goals, of the 163 patients whose serum ferritin was documented, 54 (33.1%) patients had serum ferritin 500 ng/ml. Furthermore, of 185 patients with documented TSAT, 93 (50.3%) patients had TSAT ≥ 30%.

**Table 2:** Laboratory indices recorded for the study sample

Lab test	Total (n = 226)
**Serum ferritin (ng/ml)**	
n (%)	163 (72.1)
Mean ± SD	447.49 ± 464.45
Range	4.63–2016
˂ 500	109 (66.9)
≥ 500	54 (33.1)
**TSAT (%)**	
n (%)	185 (81.9)
Mean ± SD	32.33 ± 17.16
Range	5.41–130
˂ 30	92 (49.7)
≥ 30	93 (50.3)
**C- reactive protein (mg/l)**	
n (%)	29 (12.8)
Mean ± SD	22.22 ± 50.49
Range	0.49–272
**Albumin (g/dl)**	
n (%)	182 (80.5)
Mean ± SD	3.82 ± 0.48
Range	1–5.62
**Total bilirubin (mg/dl)**	
n (%)	36 (15.9)
Mean ± SD	0.37 ± 0.15
Range	0.2–0.8
**Blood urea nitrogen (mg/dl)**	
n (%)	222 (98.2)
Mean ± SD	55.73 ± 54.85
Range	4.30–728
**Fasting blood glucose (mg/dl)**	
n (%)	109 (48.2)
Mean ± SD	140.69 ± 86.37
Range	63–683
**Serum creatinine (mg/dl)**	
n (%)	223 (98.7)
Mean ± SD	7.89 ± 3.08
Range	0.92–21.3
**Phosphorus level (mg/dl)**	
n (%)	225 (99.6)
Mean ± SD	10.71 ± 43.93
Range	0.94–441
**Calcium level (mg/dl)**	
n (%)	225 (99.6)
Mean ± SD	9.71 ± 8.12
Range	6.14–95
**Potassium level (mmol/l)**	
n (%)	224 (99.1)
Mean ± SD	4.71 ± 0.90
Range	2.70–7.40
**Sodium level (mEq/l)**	
n (%)	224 (99.1)
Mean ± SD	136.24 ± 13.07
Range	3.92–154

**Abbreviations** TSAT: Transferrin saturation, SD: Standard deviation

### Erythropoietin stimulating agent used in the study sample

The results showed that 196 (86.7%) patients used darbepoetin alfa; the mean dose administered (± SD) was 42.1 ± 16.2 mcg. Among 196 patients, 122 (62.2%) received 30 mcg, 69 (35.2%) received 60 mcg, and five (2.6%) received 90 mcg.

Of 30 patients (13.3%) who did not receive ESAs, 20% (*n* = 6) had a Hb level > 13 g/dl. However, of the 84 patients with Hb levels < 10 g/dl, 2.4% (*n* = 2) did not start ESA therapy. Among the 196 patients who received darbepoetin alfa, 24% (*n* = 47) had a Hb level > 11.5 g/dl.

### Characteristics of the study sample with differences in hemoglobin goal

There was no substantial association between the Hb goal and age category, sex, location, level of education, marital status, employment status, smoking, exercising, or family history of renal disease. Furthermore, there was no significant association between the Hb target and the number of HDs per week, the duration of renal failure, and chronic medications. On the other hand, a significant association was reported between the hemoglobin target and the total number of comorbidities (*p* = 0.045) and receiving darbepoetin alfa (*p* < 0.001) (Table 1).

### Univariate and multivariate analyses of factors associated with HRQOL

The medians (interquartile ranges) of the EQ-5D-5 L index and the EQ-VAS score were 0.69 (0.49–0.81) and 60 (50–75), respectively. Factors such as marital status, having a higher level of education, and a lower number of comorbidities were predictors of Eq. 5D5L. Participants who exercised had higher median HRQOL scores (0.82 (0.79-1.00) for Eq. 5D5L and 70.00 (70.00-89.50) for EQ-VAS) than those who did not exercise (0.66 (0.44–0.79) for Eq. 5D5L and 60.00 (50.00–70.00) for EQ-VAS). However, other variables, such as duration and frequency of hemodialysis, were not significantly associated with the two HRQOL measurement scales (Tables 3 and 4).

**Table 3:** Characteristics of the study sample according to quality of life scores

Characteristic	Total (N = 226)	EQ5D	P value	EQ-VAS	P value
	n (%)	Median [Q1-Q3]		Median [Q1-Q3]	
**Gender**			**0.012**		0.622
Male	121 (53.5)	0.73 (0.52–0.83)		60.00 (50.00–70.00)	
Female	105 (46.5)	0.64 (0.42–0.78)		60.00 (50.00-78.75)	
**Age category (years)**			0.364		0.959
< 60	113 (50.0)	0.71 (0.45–0.82)		60.00 (50.00–70.00)	
≥ 60	113 (50.0)	0.65 (0.49–0.79)		60.00 (50.00–80.00)	
**Locality**			0.188		0.56
Rural	117 (51.8)	0.69 (0.49–0.81)		60.00 (50.00–70.00)	
Urban	86 (38.1)	0.70 (0.49–0.81)		60.00 (50.00–80.00)	
Camp	23 (10.2)	0.60 (0.39–0.77)		50.00 (50.00–70.00)	
**Marital status**			**0.006**		0.077
Married	178 (78.8)	0.70 (0.53–0.82)		60.00 (50.00–75.00)	
Single	48 (21.2)	0.61 (0.32–0.77)		50.00 (46.25-70.00)	
**Level of education**			**< 0.001**		0.093
Illiterate	19 (8.4)	0.50 (0.28–0.73)		60.00 (50.00–80.00)	
Primary	80 (35.4)	0.61 (0.39–0.78)		55.00 (50.00–70.00)	
Secondary	86 (38.1)	0.70 (0.56–0.83)		60.00 (40.00-76.25)	
University	41 (18.1)	0.79 (0.68–0.86)		70.00 (60.00-78.75)	
**Employment status**			**0.001**		**0.043**
Unemployed	89 (39.4)	0.64 (0.43–0.79)		60.00 (50.00–80.00)	
Employed	13 (5.8)	0.82 (0.76–0.97)		75.00 (52.50–90.00)	
Previously employed before renal failure	124 (54.9)	0.68 (0.51–0.79)		60.00 (50.00–70.00)	
**Smoking**			0.209		0.555
Current smoker	52 (23)	0.75 (0.59–0.82)		60.00 (50.00–70.00)	
Previous smoker	38 (16.8)	0.67 (0.45–0.83)		60.00 (50.00–80.00)	
Nonsmoker	136 (60.2)	0.65 (0.44–0.79)		60.00 (50.00–70.00)	
**Exercise**			**< 0.001**		**0.001**
No	209 (92.5)	0.66 (0.44–0.79)		60.00 (50.00–70.00)	
yes	17 (7.5)	0.82 (0.79-1.00)		70.00 (70.00-89.50)	
**Family history**			0.543		0.463
No	187 (82.7)	0.69 (0.46–0.80)		60.00 (50.00–75.00)	
Yes	39 (17.3)	0.69 (0.54–0.82)		60.00 (50.00–70.00)	
**Herbal use**			**< 0.001**		0.116
No	103 (45.6)	0.61 (0.38–0.77)		60.00 (50.00–70.00)	
Yes	123 (54.4)	0.73 (0.56–0.83)		60.00 (50.00–80.00)	
**Total number of medications**			0.745		0.854
< 7	115 (50.9)	0.67 (0.44–0.81)		60.00 (50.00–75.00)	
≥ 7	111 (49.1)	0.69 (0.50–0.80)		60.00 (50.00-71.25)	
**Number of dialysis per week**			0.659		0.674
< 3	25 (11.1)	0.67 (0.41–0.78)		60.00 (50.00-77.50)	
≥ 3	201 (88.9)	0.69 (0.49–0.81)		60.00 (50.00–70.00)	
**Duration of renal failure**			0.219		0.494
< 5	124 (54.9)	0.72 (0.51–0.81)		60.00 (50.00–80.00)	
5-<10	72 (31.9)	0.65 (0.51–0.81)		60.00 (50.00–70.00)	
≥ 10	30 (13.3)	0.62 (0.31–0.77)		60.00 (40.00–70.00)	
**Years of hemodialysis**			0.095		0.183
< 4	146 (64.6)	0.71 (0.55–0.81)		60.00 (50.00–80.00)	
≥ 4	80 (35.4)	0.62 (0.42–0.79)		60.00 (41.25-70.00)	
**Hours of hemodialysis**			0.613		0.489
< 4	158 (69.9)	0.67 (0.48–0.80)		60.00 (50.00–70.00)	
≥ 4	68 (30.1)	0.71 (0.50–0.81)		60.00 (45.00–80.00)	
**Number of comorbid diseases**			**0.017**		0.082
≤ 2	32 (14.2)	0.73 (0.45–0.84)		60.00 (50.00–70.00)	
3–4	97 (42.9)	0.73 (0.57–0.82)		60.00 (50.00–80.00)	
5–6	75 (33.2)	0.63 (0.41–0.77)		50.00 (45.00–70.00)	
> 6	22 (9.7)	0.57 (0.25–0.83)		60.00 (38.75-75.00)	
**Received darbepoetin alfa**			0.874		0.341
No	30 (13.3)	0.69 (0.51–0.82)		60.00 (50.00–82.00)	
Yes	196 (86.7)	0.69 (0.48–0.80)		60.00 (50.00–70.00)	
**Hemoglobin level**			**0.039**		**0.004**
< 11	134 (59.3)	0.65 (0.42–0.79)		50.0 (43.8–70.0)	
≥ 11	92 (40.7)	0.73 (0.51–0.82)		60.0 (50.0-78.8)	

**Table 4:** Predictors of Eq. 5D using regression analysis

	Model	Unstandardized Coefficients		Standardized Coefficients	t	p value*	95.0% Confidence Interval for B		Collinearity Statistics
		B	Std. Error	Beta			Lower Bound	Upper Bound	VIF
1	**(Constant)**	0.683	0.122		5.609	0.000	0.443	0.923	
	**Gender**	− 0.021	0.037	− 0.042	− 0.567	0.571	− 0.095	0.052	1.538
	**Marital Status**	− 0.099	0.039	− 0.162	-2.501	**0.013**	− 0.176	− 0.021	1.154
	**Level of education**	0.049	0.019	0.172	2.657	**0.008**	0.013	0.086	1.159
	**Employment status**	− 0.008	0.018	− 0.032	− 0.476	0.635	− 0.043	0.027	1.288
	**Exercise**	0.216	0.058	0.228	3.698	**< 0.001**	0.101	0.331	1.053
	**Herbal use**	0.079	0.031	0.157	2.540	**0.012**	0.018	0.140	1.054
	**Number of comorbid diseases**	− 0.044	0.018	− 0.148	-2.362	**0.019**	− 0.080	− 0.007	1.084
	**Hemoglobin level**	0.051	0.031	0.100	1.623	0.106	− 0.011	0.113	1.056

a. Dependent variable: EQ-5D-5 L index value

The median (interquartile range) of the EQ-5D-5 L index for patients with a Hb level < 11 was 0.65 (0.42–0.79), while it was 0.73 (0.51–0.82) for those who had a Hb level ≥ 11. Furthermore, the median EQ-VAS score for patients with Hb levels < 11 was 50.0 (43.8–70.0) compared to 60.0 (50.0-78.8) for patients with Hb levels ≥ 11. The hemoglobin level was found to significantly affect both scales of quality of life: EQ-5D-5 L (*p* = 0.039) and EQ-VAS (*p* = 0.004). According to the multivariate analysis, a low level of hemoglobin was the predictor of poor quality of life on the EQ-VAS scale only (Table 5).

**Table 5:** Predictors of EQ-VAS using regression analysis

	Model	Unstandardized Coefficients		Standardized Coefficients	t	p value*	95.0% Confidence Interval for B		Collinearity Statistics
		B	Std. Error	Beta			Lower Bound	Upper Bound	VIF
1	**(Constant)**	57.996	3.589		16.161	0.000	50.923	65.068	
	**Employment status**	-1.125	1.443	− 0.050	− 0.780	0.436	-3.968	1.718	1.007
	**Exercise**	16.990	5.246	0.209	3.239	**0.001**	6.652	27.328	1.008
	**Hemoglobin level**	8.673	2.815	0.199	3.081	**0.002**	3.126	14.220	1.007

Dependent variable: Visual analog scale

## Discussion

Few studies have been established to study the treatment of anemia among HD patients and its association with HRQOL [15, 34]. Therefore, this work is the first in Palestine to provide baseline data and information that help to assess anemia management in HD patients and its association with HRQOL and patient characteristics.

The sociodemographic findings of the current study were relatively close to the results of a previous publication in Palestine in 2015, such as age, sex, duration of HD and smoking status [35]. In the current study, most patients had three weekly dialysis sessions with a median length of 3.5 h for each session. Comparable findings were observed in a study conducted in Palestine that aimed to define variables that influence the quality of life of HD patients [32].

Al-Ramahi et al. [35] found that hypertension was the most prevalent comorbidity (78.5%), followed by DM (42.5%), among patients with HD. Furthermore, the number of medications used by patients ranged from one to fifteen, with a mean (± SD) of 7.87 ± 2.44. Calcium carbonate (77.1%) and alfacalcidol (73.8%) were the most prescribed medications. All the results mentioned were comparable to our findings.

In our study, the mean Hb level (10.63 g/dl) was lower than that of a Saudi Arabia study (11.16 g/dl) [17] and slightly higher than that of a Lebanon study (10.29 g/dl) [14]. By comparing our findings with research conducted in Palestine. The mean level of Hb was 8.84 ± 1.52 g/dl, and according to NKF-KDOQI, the Hb goals were only achieved in 8.9% of the patients, and 43% had Hb between 9 and 11.5 g/dl. ESA is regularly initiated when the Hb level is between 9 and 10 g/dl, as recommended by KDIGO, and it is suggested not to maintain Hb > 11.5 g/dl. Increasing Hb > 11.5–13 g/dl in HD patients may be associated with increased impairments, such as an increased risk of thrombosis in vascular access, stroke, and hypertension [36, 37]. In this analysis, 37.16% of the patients had Hb levels < 10 g/dl, and 2.4% did not initiate ESA. Furthermore, 24% of those receiving ESA had Hb levels > 11.5 g/dl, which was against the KDIGO recommendation.

However, the current study reported that the mean dose (SD) of darbepoetin alfa administered to HD patients was 42.1 ± 16.2 mcg, with the majority receiving the 30 mcg dose. Furthermore, a high percentage of patients (76%) who received ESA had a Hb level ≤ 11.5 g/dl. This could be due to hyporesponsiveness to ESA. Several factors influence the hyporesponsiveness of ESA, including noncompliance, iron deficiency, hyperparathyroidism, inflammation, and bone marrow disorder [38].

According to the NKF-KDOQI and KDIGO recommendations on achieving serum ferritin > 200 ng/ml and > 500 ng/ml, 57.6% and 46.8% of our sample had achieved these goals, respectively. Importantly, serum ferritin and TSAT were available for 72.1% and 81.9% of the patients, respectively. The mean serum ferritin concentration (SD) was 447.49 ± 464.45 ng/ml, while the mean TSAT was 32.33 ± 17.16%. These values were lower than those previously found, 693 ± 420.5 ng/ml for serum ferritin and 38 ± 19.7 for TSAT [17].

Iron administration in patients with HD is frequent. Therefore, iron status is mandatory, as it contributes to anemia and hyporesponsiveness to ESA therapy. However, notable differences were found in the percentage of patients who received iron supplementation and ESA between our results and the findings of the previous Palestinian study, which were 69.9% and 5.1%, respectively [19]. The fluctuation between both studies may be that the previous study was conducted for a period when PMOH could not provide ESA therapy and was performed in one dialysis center. However, another study showed that 81.6% of patients received iron supplementation intravenously [17].

Our results showed an insignificant association between Hb level and any documented demographic characteristics. A previous study from Saudi Arabia found that Hb levels were not significantly associated with age. However, the females had a statistically significant low Hb level [17]. Furthermore, a study conducted in Iran aimed to assess compliance with KDIGO guidelines and evaluate the relationship between Hb and morbidity [39] showed that younger age was associated with hemoglobin levels lower than 10 g/dL. On the other hand, a study that enrolled 169 patients for 12 months in a single center in Turkey found that Hb variability is positively correlated with age [40]. On the other hand, the current study did not show a significant association between Hb level and years of dialysis, similar to Al-Ageel et al. [17].

The present data revealed that the medians (interquartile ranges) of the EQ-5D-5 L index and the EQ-VAS score were 0.69 (0.49–0.81) and 60 (50–75), respectively. The results of the EQ-5D-5 L index and the EQ-VAS score of a study conducted in Palestine were 0.41 (0.06–0.77) and 50 (50–70), respectively [32]. This difference might be due to the differences in the centers included in both studies.

This study found a significant association between quality of life and hemoglobin targets. A similar finding was found in a study carried out in Iran [16], which showed a significant correlation between Hb and HRQOL. Additionally, a cross-sectional study investigated seven centers in Canada and the United States during 2003–2006 [41] revealed that patients with an increased level of Hb of 11–13 g/dl significantly improved their quality of life. Regression analysis found that a low Hb level significantly lowers HRQOL on the EQ-VAS scale but not on the Eq. 5D5L. A cross-sectional study conducted in 2007 in western China investigated HRQOL among hemodialysis patients using another version of the EQ-5D (EQ-5D-3 L), which has three levels of response, and showed an insignificant association between the EQ-5D score and the hemoglobin level [42]. Fatigue, shortness of breath, headache, reduced mental state, and insomnia are nonspecific anemia symptoms resulting from decreased oxygen supply to body tissues and organs [43–45]. These symptoms can affect the patient’s well-being and quality of life. Furthermore, other serious consequences, such as cardiovascular complications, may develop because of anemia progression in CKD patients [43, 44].

### Strengths and limitations

This research is the first in Palestine to study the treatment of anemia in patients with HD and determine its association with the HRQOL, demographics, and clinical characteristics of patients. Furthermore, the data were collected through face-to-face interviews and patient medical records, providing reliable and comprehensive data. However, our study had some limitations. First, it was a cross-sectional study, making the reason and the outcome unclear and the resulting associations difficult to interpret. For instance, we cannot determine whether anemia causes poor quality of life or if a low quality of life can worsen anemia. Second, the patients were chosen using a convenience sampling technique, which can disturb the generalizability of the study findings. Third, although face-to-face interviews provide accurate screening, offer the capture of verbal and nonverbal questions that show the level of discomfort with the question and capture the behavior and emotions, bias could be announced. Last, our study did not determine some clinical variables, such as parathyroid hormone level, infection, inflammation, and certain blood factors that may affect Hb level or ESA hyporesponsiveness. This means that multiple factors are associated with the Hb level, and we recommend studying all clinical and sociodemographic variables that have significant correlations with the Hb level; therefore, we could increase the validity of the findings.

## Conclusions

Our study found that more than half of the patients had a Hb level below the recommended KDIGO target. Approximately half of the patients received iron replacement supplementation, and most of them received ESA therapy. Although they received ESA, many patients had a high Hb level below the target. Consequently, we recommend choosing the most desirable, cost-effective, and optimal therapy and regularly following up with an evaluation of variables that are associated with an inadequate response to ESA. In addition, the study found a significant association between patients’ Hb levels and HRQOL. Therefore, the appropriate management of anemia in HD patients should be followed by adherence to guideline recommendations, consequently improving quality of life.

## Electronic supplementary material

Below is the link to the electronic supplementary material.

Supplementary Material 1

## Data Availability

Data and materials used in this work are available from the corresponding author upon request. This manuscript is a component of a master’s thesis in clinical pharmacy at the Faculty of Graduate Studies, An-Najah National University. The thesis was published as part of self-archiving in institutional repositories (university repository: https://repository.najah.edu/items/d4a58c20-2cbc-4e50-9c10-f902ecbb9949).

## List of abbreviations

HD: Hemodialysis
ESA: Erythropoietin stimulating agents
HRQOL: Health-related quality of life
EQ-5D-5 L: The 5-Dimension European Quality of Life scale
EQ-VAS: Visual analog scale
Hb: Hemoglobin
KDIGO: Kidney Disease Improving Global Outcomes:ESRD:End-stage renal disease
CKD: Chronic kidney disease
RBCs: Red blood cells
LVH: Left ventricular hypertrophy
TSAT: Transferrin saturation
ESAM 2003: The European Survey of Anemia Management 2003
CRP: C-reactive protein
NKF-KDOQI: National Kidney Foundation-Kidney Disease Outcomes Quality Initiative
DM: Diabetes mellitus
QOL: Quality of life
IRB: Institutional Review Board
PMOH: Palestinian Ministry of Health
SPSS: Statistical Package for Social Sciences
