# Supplementary material for: A multicenter descriptive analysis of anemia management in hemodialysis patients and its association with quality of life
Source: BMC Nephrol. 2023 Jun 30;24:197. doi: 10.1186/s12882-023-03254-7 (PMC10314542; doi:10.1186/s12882-023-03254-7)
Supplement: Supplementary file 1 — Supplementary Material 1 [file 12882_2023_3254_MOESM1_ESM.doc]

**Supplementary Tables 1-3.** **Supplementary Table 1:** Comorbid diseases among the study sample; **Supplementary Table 2:** Herbal remedies and nutrients used by the study sample; **Supplementary Table 3:** Chronic medications used by the study sample.

**Supplementary Table 1** Comorbid diseases among the study sample

| **Co-morbid disease** | **Total (n = 226)**  **n (%)** |
| --- | --- |
| Hypertension | 198 (87.6) |
| Diabetes Mellitus | 127 (56.2) |
| Ischemic heart disease | 96 (42.5) |
| Heart failure | 61 (27) |
| Hyperparathyroidism | 48 (21.2) |
| Dyslipidemia | 40 (17.7) |
| Gout | 38 (16.8) |
| Retinopathy | 26 (11.5) |
| Osteoarthritis | 21 (9.3) |
| Nephrotoxicity | 20 (8.8) |
| Urinary tract infection | 19 (8.4) |
| Urinary stones | 15 (6.6) |
| Hypothyroidism | 12 (5.3) |
| Polycystic kidney disease | 10 (4.4) |
| Atrophic kidney | 9 (4) |
| Atrial fibrillation | 7 (3.1) |
| Benign Prostatic Hypertrophy (BPH) | 6 (2.7) |
| Asthma | 5 (2.2) |
| Gastroesophageal Reflux Disease (GERD) | 4 (1.8) |
| Osteoporosis | 4 (1.8) |
| Chronic Obstructive Pulmonary Disease (COPD) | 3 (1.3) |
| Hepatitis | 3 (1.3) |
| Glaucoma | 3 (1.3) |
| Familial Mediterranean Fever (FMF) | 2 (0.9) |
| Meniere’s syndrome | 2 (0.9) |
| Systemic infection | 1 (0.4) |
| Rheumatoid arthritis | 1 (0.4) |
| Systemic Lupus Erythematosus (SLE) | 1 (0.4) |
| Glomerular Nephritis | 1 (0.4) |
| Crohn’s disease | 1 (0.4) |
| Psoriasis | 1 (0.4) |
| Alport syndrome | 1 (0.4) |
| Hypoparathyroidism | 1 (0.4) |

**Supplementary Table 2** Herbal remedies and nutrients used by the study sample

| **Herbs** | **Total (n = 226)**  **n (%)** |
| --- | --- |
| *Acacia senegal* (Gum acacia) | 71 (31.4) |
| Mixed herbs | 42 (18.6) |
| *Carum petroselinum* (Parsley) | 18 (8) |
| *Pimpinellaanisum* L. (Anise) | 16 (7.1) |
| *Matricariachamomilla* L. (Chamomile) | 11 (4.9) |
| Honey | 11 (4.9) |
| *Trigonella foenum graecum* (Fenugreek) | 10 (4.4) |
| *Hordeum vulgare* (Barely) | 10 (4.4) |
| *Rosmarinus officinalis* L. (Rosemary) | 7 (3.1) |
| *Verbena officinalis* (Vervain) | 4 (1.8) |
| *Nigella sativa* (Black caraway) | 3 (1.3) |
| *Salvia officinalis* L. (Sage) | 3 (1.3) |
| *Boswellia serrata* (Frankincense) | 2 (0.9) |
| *Zingiber officinale Roscoe (*Ginger) | 2 (0.9) |
| *Foeniculum vulgare* (Fennel) | 1 (0.4) |
| *Menthapiperita* L. (Peppermint) | 1 (0.4) |
| *Tilia americana* (Lindens) | 1 (0.4) |
| *Thymus vulgaris* L. (Thyme) | 1 (0.4) |
| *Raphanus sativus* (Radish) | 1 (0.4) |
| *Crocus sativus L.* (Saffron) | 1 (0.4) |
| *Urtica dioica. L.* (Nettles) | 1 (0.4) |
| Kefir | 1 (0.4) |
| *Anabasis aphylla L.* (Anabasis) | 1 (0.4) |
| *Teucrium polium L.* (Germander) | 1 (0.4) |

**Supplementary Table 3** Chronic medications used by the study sample

| **Medication** | **Total (n = 226)**  **n (%)** |
| --- | --- |
| Darbepoetin  Dose (mcg):  mean ± SD  30 mcg  60mcg  90mcg | 196, (86.7)  42.1 ± 16.2  122 (62.2)  69 (35.2)  5 (2.6) |
| Calcium carbonate | 193 (85.4) |
| Alfacalcidol | 190 (84.1) |
| Iron III- hydroxide sucrose (IV)  Dose (mg) | 128 (56.6)  100 mg |
| Aspirin | 102 (45.1) |
| Amlodipine | 80 (35.4) |
| Ranitidine | 75 (33.2) |
| Insulin | 66 (29.2) |
| Atorvastatin | 66 (29.2) |
| Furosemide | 64 (28.3) |
| Sevelamer | 55 (24.3) |
| Paracetamol | 42 (18.6) |
| Bisoprolol | 41 (18.1) |
| Allopurinol | 29 (12.8) |
| Clopidogrel | 26 (11.5) |
| Isosorbide mononitrate | 23 (10.2) |
| Omega 3 | 18 (8) |
| Enalapril | 17 (7.5) |
| Enoxaparin | 16 (7.1) |
| Carvedilol | 13 (5.8) |
| Doxazosin | 12 (5.3) |
| Atenolol | 11 (4.9) |
| L- Thyroxin | 11 (4.9) |
| Esomeprazole | 9 (4) |
| Folic acid | 9 (4) |
| Nifedipine | 8 (3.5) |
| Warfarin | 8 (3.5) |
| Loratadine | 8 (3.5) |
| Prednisolone | 4 (1.8), Total (n= 225) |
| Valsartan | 4 (1.8) |
| Hydralazine | 4 (1.8) |
| Omeprazole | 4 (1.8) |
| Colchicine | 4 (1.8) |
| Amlodipine / Valsartan | 3 (1.3) |
| Glimepiride | 3 (1.3) |
| Ipratropium Bromide | 3 (1.3) |
| Betahistine | 3 (1.3) |
| Cinacalcet | 3 (1.3) |
| Sodium valproate | 3 (1.3) |
| Phenytoin | 3 (1.3) |
| Labetalol | 2 (0.9) |
| Amiodarone | 2 (0.9) |
| L-Dopa | 2 (0.9) |
| Midodrine | 2 (0.9) |
| Vildagliptin | 2 (0.9) |
| Tamsulin | 2 (0.9) |
| Carbamazepine | 1 (0.4), Total (n= 225) |
| Lercanidipine | 1 (0.4) |
| Sacubitril / Valsartan | 1 (0.4) |
| Metolazone | 1 (0.4) |
| Metformin | 1 (0.4) |
| Saxagliptin | 1 (0.4) |
| Glibenclamide | 1 (0.4) |
| Pantoprazole | 1 (0.4) |
| Vitamin B-12 | 1 (0.4) |
| Chlorpheniramine | 1 (0.4) |
| Montelukast | 1 (0.4) |
| Alfuzosin | 1 (0.4) |
| Bezafibrate | 1 (0.4) |
| Bisacodyl | 1 (0.4) |
| Gabapentin | 1 (0.4) |
| Pregabalin | 1 (0.4) |
| Clonazepam | 1 (0.4) |

**Abbreviation:** IV: intravenous, SD: standard deviation
